# Resistin as a Systemic Inflammation-Related Biomarker for Sarcopenia in Patients With Chronic Obstructive Pulmonary Disease

**DOI:** 10.3389/fnut.2022.921399

**Published:** 2022-07-12

**Authors:** Jinghan Gao, Mingming Deng, Yanxia Li, Yan Yin, Xiaoming Zhou, Qin Zhang, Gang Hou

**Affiliations:** ^1^Department of Pulmonary and Critical Care Medicine, First Hospital of China Medical University, Shenyang, China; ^2^Department of Pulmonary and Critical Care Medicine, Center of Respiratory Medicine, China-Japan Friendship Hospital, Beijing, China; ^3^Graduate School of Peking Union Medical College, Chinese Academy of Medical Sciences, Beijing, China; ^4^National Center for Respiratory Medicine, Beijing, China; ^5^Institute of Respiratory Medicine, Chinese Academy of Medical Sciences, Beijing, China; ^6^National Clinical Research Center for Respiratory Diseases, Beijing, China; ^7^Respiratory Department, The First Affiliated Hospital of Dalian Medical University, Dalian, China; ^8^Department of Pulmonary and Critical Care Medicine, Disease, Fuwai Hospital, Peking Union Medical College, Chinese Academy of Medical Sciences, Beijing, China

**Keywords:** chronic obstructive pulmonary disease, sarcopenia, resistin, TNF-α, biomarker

## Abstract

**Background:**

Sarcopenia is common in patients with chronic obstructive pulmonary disease (COPD) and is mainly caused by systemic inflammation. Resistin acts as a proinflammatory cytokine and is involved in the activation of multiple inflammatory signaling pathways. The aim of this study was to determine the relationship between resistin levels and systemic inflammation and to assess the clinical value of circulating resistin for sarcopenia in patients with COPD.

**Methods:**

In this prospective observational study, we enrolled 235 patients with COPD who were divided into development and validation sets. The definition of sarcopenia followed the guidelines from the Asian Working Group for Sarcopenia. Serum concentrations of resistin and TNF-α were measured using an enzyme-linked immunosorbent assay (ELISA).

**Results:**

In this study, higher serum resistin levels were significantly associated with lower skeletal muscle mass and muscular strength. The serum resistin levels in patients with sarcopenia were significantly higher than those in patients without sarcopenia. The serum resistin level had positive correlations with the serum TNF-α level (*r* = 0.250, *p* = 0.007). The predictive efficacy of the serum resistin level (AUC: 0.828) for sarcopenia was superior to that of the serum TNF-α level (AUC: 0.621). The cutoff point (7.138 ng/ml) for the serum resistin level was validated in the validation set (AUC: 0.818).

**Conclusions:**

Serum resistin levels were associated with systemic inflammation and can be used accurately and easily to predict sarcopenia in patients with COPD.

## Introduction

Chronic obstructive pulmonary disease (COPD) is a chronic airway disease characterized by persistent respiratory symptoms and limited airflow that is often accompanied by multiple comorbidities involving the cardiovascular system, digestive system, hematologic system, musculoskeletal system, etc. (1, 2). Among the comorbidities, sarcopenia is associated with a poor prognosis and increased hospitalization and mortality rates (3). Patients with COPD have an increased risk of developing sarcopenia, with prevalence estimates ranging from 15 (4) to 55% (5). Sarcopenia had a consistently negative impact on a range of COPD-related clinical outcomes, including exercise capacity, balance, quadriceps, and handgrip strength, and physical activity levels (6–9). It was also associated with increased symptom burden and poorer quality of life. Early identification and intervention of sarcopenia is important to improve the quality of life and prognosis of patients with COPD. However, the diagnosis of sarcopenia requires simultaneous assessment of muscle mass, muscle strength, and physical function (10, 11). Therefore, it is time-consuming and difficult to assess sarcopenia in all COPD patients in clinical practice.

Inflammation and oxidative stress play a key role in the decline in respiratory function in COPD (12). More commonly, in patients with COPD, cigarette smoking-induced inflammation and oxidative stress in the lungs “spill over” into the circulation, manifesting as systemic inflammation (2). Persistent systemic inflammation is associated with an accelerated decline in lung function and is exacerbated during exacerbation episodes. Systemic inflammation is now a recognized risk factor for other complications commonly seen in patients with COPD (13). Several studies (14, 15) have suggested a possible association between systemic inflammation and comorbid sarcopenia in patients with COPD. TNF-α are cytokines related to chronic inflammatory metabolic disorders that can result in a decrease in protein synthesis in skeletal muscle and associated with a decrease in muscle mass and strength (16). Injections of exogenous TNF-α decreased muscle mass and its ability to regenerate in mouse model (17). Previous study suggested that sarcopenia is associated with increased TNF-α levels (18). Systemic ablation of TNF-α prevented sarcopenia (19). These results suggest that TNF-α may play an important role in the development of sarcopenia. Previous study in COPD shown that serum TNF-α levels are significantly associated with grip strength and skeletal muscle mass, which are important determinants of sarcopenia in patients with stable COPD (20, 21). These results suggest that systemic inflammation-related factors may be important biomarkers for patients with COPD in predicting sarcopenia.

Resistin was originally described as an adipokine causing insulin resistance in mice that is produced by white adipose tissue (22). In humans, resistin also acts as a proinflammatory cytokine that is produced mainly by cells of the inflammatory system, such as monocytes, macrophages, and neutrophils (23). Previous studies have shown that resistin is an endogenous ligand for Toll-like receptor 4 (TLR4) and is involved in the activation of multiple inflammatory signaling pathways (24, 25). Resistin could disrupts myotube and nuclear fusion by activating the classical NF-κB pathway (26). In additional, resistin inhibits myogenic myoblast differentiation (27). Resistin has been shown to impair insulin signaling (28), which is an important regulator mediating muscle growth and regeneration (29). These results suggest that resistin may play an important role in the development process of sarcopenia. A previous study (30) indicated that the mean resistin levels in patients with sarcopenia were 2-fold higher than those in healthy controls. In addition, significant negative correlations have been shown between the forced expiratory volume in 1 second (FEV_1_) and resistin levels in patients with COPD (30). The serum resistin level is associated with osteoporosis in patients with COPD (31). The accuracy of circulating resistin levels in predicting sarcopenia in patients with COPD has not been established.

The aim of this study was to assess the clinical value of circulating resistin in prospectively screening for sarcopenia in patients with COPD and to determine the cutoff value for use in clinical practice.

## Materials and Methods

### Study Design and Subjects

This is a cross-sectional analysis that starts from a longitudinal study and was conducted at the First Hospital of China Medical University and the First Hospital of Dalian Medical University from August 2018 to December 2019. Patients with COPD who met the Global Initiative for Chronic Obstructive Lung Disease (GOLD) criteria were eligible for inclusion (32), and the predicted post-bronchodilator FEV_1_% was divided into ≥80%, 50–79%, 30–49%, and <30% according to GOLD stage. The exclusion criteria were as follows: acute exacerbation within the past 1 month; presence of severe cardiovascular disease, active lung disease, patients with edema or fluid overload, etc.; long-term systemic hormone therapy; and inability to read or understand the informed consent. The Medical Ethics Committee of the First Hospital of China Medical University approved this study (No. 2018-144-2), and all patients provided written informed consent. We included all patients with stable chronic obstructive pulmonary disease in the outpatient clinic who met the inclusion and exclusion criteria and who signed an informed consent during August 2018 to December 2019. The estimated total sample size used for univariate and multivariate logistic analyses to explore the clinical factors associated with sarcopenia in patients with COPD followed previous studies (33, 34). It was used for univariate and multivariate logistic analyses to explore the clinical factors associated with sarcopenia in patients with COPD. Given the prevalence of sarcopenia in patients with stable COPD of 25% (20) and 5 covariates in the logistic model, the sample size was estimated to be 200 according to the equation (*N* = 10 k/p) (33, 34). The final sample size was composed of 235 people. Diabetes was present in 43 (18.30%) patients. Hypertension was present in 56 (23.83%) patients. To obtain the desired level of statistical power for evaluating the accuracy of the diagnostic test, the minimal sample size required was calculated based on the estimated sensitivity and specificity (35):


$$\begin{array}{l} N_{Se}=\frac{Z\frac{\alpha^{2}}{2}\times \hat{Se}\times \left(1-\hat{Se}\right)}{d^{2}\times Prev};N_{Sp}=\frac{Z\frac{\alpha^{2}}{2}\times \hat{Sp}\times \left(1-\hat{Sp}\right)}{d^{2}\times \left(1-Prev\right)}; \end{array}$$

We estimated the sensitivity (Se) and specificity (Sp) for the serum resistin level to diagnose sarcopenia as 90 and 70%, respectively, according to our preliminary study; we estimated the prevalence of sarcopenia in patients with COPD as ~30% according to data from this study. For a maximum marginal error of the estimate not exceeding 10% with a 95% confidence level. The total required sample size was 115 patients which calculated using sensitivity. And, the total required sample size was 115 patients which calculated using specificity. Therefore, the total required sample size was 115 patients. A total of 117 patients with COPD who were recruited from the First Hospital of China Medical University were enrolled in the development set to assess the clinical value of resistin and determine the cutoff values. Another 118 patients with COPD who were recruited from the First Hospital of Dalian Medical University were enrolled in the validation set. And, the number of participants in development set and validation set is adequate to know the resistin cutoff point.

### Data Collection and Measurements

Age, sex, smoking status, and smoking pack-years were recorded. Spirometry measurements were performed as recommended by the American Thoracic Society and the European Respiratory Society guidelines using a Jaeger MasterScreen system (Viasys Healthcare GmbH, Hoechberg, Germany). Dyspnea and health status were assessed based on the Chinese version of the mMRC dyspnea score (36) and the CAT (37). Exercise capacity was determined using the 6-min walking test following the 2002 American Thoracic Society (ATS) guidelines (38). In short, a closed, long, and straight 30-m corridor was selected indoors, and the patient was told to walk as far as possible. Standard language was used to encourage the patient. After 6 min, the patient was told “time is up” and asked to stop walking. The testing personnel recorded the distance traveled in meters. Venous blood was obtained in the fasted state. Blood samples were collected into serum separator tubes, left to stand for 2 h at room temperature to clot, and then centrifuged at 3,000 rpm for 15 min at room temperature. The supernatant liquid was obtained and kept in a −80°C refrigerator. TNF-α was assessed by high-sensitivity ELISA kits (#HSTA00E; R&D, Minneapolis, MN, USA) following the manufacturer's instructions. The resistin level was determined using a human resistin Quantikine ELISA kit (#DRSN00; R&D, Minneapolis, MN, USA) according to the manufacturer's instructions.

### Measurement Thickness and Cross-Sectional Area of the Rectus Femoris

Measurement of quadriceps rectus femoris thickness and cross-sectional area followed previous studies (39, 40). Grayscale ultrasound was used with a 4- to 15-MHz linear-array transducer (SuperSonic Imagine, Aix-en-Provence, France). Two physicians from ultrasonography performed the ultrasound examinations. The patient requested refrain from strenuous exercise for 72 h prior to the test, rested quietly for 30 min and then lay on the back on the operating bed to relax the muscles of the whole body. The researchers set up a bracket to fix the ultrasound probe to reduce muscle deformation due to external forces and placed the ultrasound probe perpendicular to the patient's dominant leg. The transducer was positioned perpendicular to the long axis of the dominant leg (precisely at 3/5 of the distance from the anterior superior iliac spine to the superior patellar border). The scanning depth was set to where the femur could be discerned for orientation. Gentle contraction-relaxation maneuveres were employed to delineate muscle septa prior to image acquisition. RF_thick_ and RF_csa_ were calculated after the inner echogenic line of the rectus femoris was outlined by a movable cursor on a frozen image. RF_thick_ and RF_csa_ were taken as averages of three consecutive measurements within 10%.

### Assessment of Sarcopenia

The definition of sarcopenia followed the guidelines from the AWGS (11), as follows: low muscle mass [bioelectrical impedance (males, <7.0 kg/m^2^; females, <5.7 kg/m^2^)]; low muscle strength [handgrip strength (males, <28 kg; females, <18 kg)]; and/or poor physical performance [5-time chair stand test (5STS), ≥12 s]. Muscle mass was evaluated using bioelectrical impedance analysis [BIA] (InBody770; InBody, Seoul, South Korea). Muscle strength was assessed by handgrip strength using a JAMAR®Plus^+^ hand dynamometer (Sammons Preston, Bolingbrook, IL, USA). The 5STS was performed according to the procedure described in a previous study (41). Specifically, the patient sits on a chair (48 cm in height) without armrests, with feet on the ground, the back supported by the back of the chair, and hands folded in front of the chest. After hearing the test start command, the participant was asked to complete 5 standing and sitting movements as fast as possible; the time was recorded. During the test, the patient was instructed to cross the hands on the chest without separation. The knee joints were an equal distance from the ground when in the standing position. The participant was given verbal encouragement during the test. The test was performed 3 times, with 1-min rest intervals. The average of 3 test times was taken as the result.

### Statistical Analyses

Statistical analyses were performed using SPSS 13.0 software (IBM, Armonk, NY, USA). To check the distribution status of serum resistin levels, the Shapiro–Wilk test, histograms, and Q-Q (quantile–quantile) plots were used. Pearson's correlation coefficient was calculated to determine the association between continuous variables. Univariate and multivariate logistic analyses were used to explore the clinical factors associated with sarcopenia. Results were presented as OR and 95%CI (42). *P*-values < 0.05 were considered statistically significant. Variables with a *P*-value of < 0.05 in the univariate logistic analysis were included in the multivariate logistic to identify corresponding risk factors. For the continuous variables, differences between two groups were assessed using a *t*-test (normally distributed data) or the Mann–Whitney test (non-normal distribution). *P*-values < 0.05 were considered statistically significant.

## Results

### Baseline Characteristics

The baseline characteristics of patients with COPD are shown in Table 1.

**Table 1:** Baseline characteristics of subjects.

**Variable**	**Development set**	**Validation set**	***P-*value**
	**(*n* = 117)**	**(*n* = 118)**	
**Demographics**			
Age, years	64.9 ± 12.0	63.9 ± 9.2	0.110
Sex, m/f	83/34	77/41	0.349
**Pulmonary function**			
FEV_1_, L	1.6 ± 0.6	1.5 ± 0.5	0.370
FEV_1_, %predicted	59.8 ± 21.0	56.2 ± 20.7	0.368
FVC, L	2.8 ± 0.9	2.7 ± 0.8	0.452
FVC, % predicted	82.5 ± 23.7	84.5 ± 20.0	0.700
FEV_1_/FVC, %	55.2 ± 9.9	53.1 ± 11.1	0.232
GOLD stage			0.986
1	19	21	
2	59	57	
3	30	31	
4	9	9	
**Physical function**			
6MWD, m	369.0 ± 74.2	359.1 ± 78.1	0.370
5STS, s	7.8 ± 3.3	7.4 ± 2.3	0.624
**Body composition**			
BMI, kg/m^2^	23.9 ± 3.9	23.6 ± 3.8	0.532
Body fat (%)	28.9 ± 6.3	29.0 ± 6.4	0.907
FFM (kg)	47.0 ± 9.3	45.0 ± 9.1	0.148
FFMI (kg/m^2^)	16.84 ± 2.4	16.7 ± 2.3	0.750
SMM (kg)	17.7 ± 4.4	15.8 ± 4.6	0.137
SMMI (kg/m^2^)	6.4 ± 1.1	5.9 ± 1.4	0.143
HGS (kg)	26.6 ± 8.1	27.0 ± 1.1	0.787

### Relationships of the Serum Resistin Level With the Clinical Features of Patients With COPD

**Figure 1:**
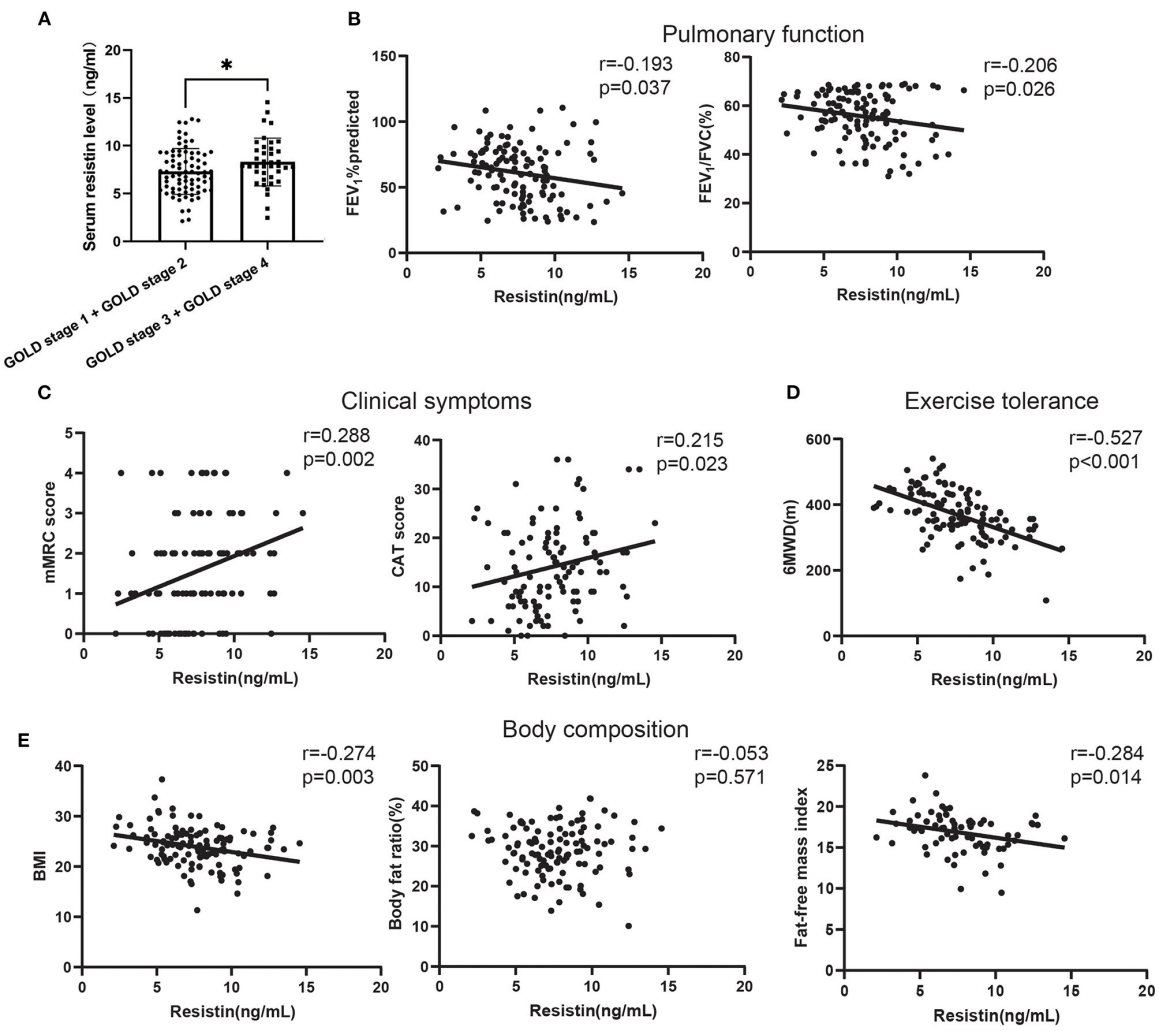
The relationships of serum resistin levels with the clinical features of patients with COPD. **(A)** Difference in serum resistin level in patients with global initiative for chronic obstructive lung disease (GOLD) A and GOLD B and in patients with GOLD C and GOLD D; **(B)** The relationship between serum resistin level and FEV_1_%predicted, FEV_1_/FVC; **(C)** The relationship between serum resistin level and mMRC score, CAT score; **(D)** The relationship between serum resistin level and 6MWD; **(E)** The relationship between serum resistin level and BMI, body fat ratio, FFMI. ^*^p < 0.05.

First, the results (Supplementary Figure 1) from histograms, Q-Q plots, and Shapiro–Wilk's test suggested that serum resistin expression data obeyed a normal distribution (*p* = 0.429). Next, to verify the clinical value of resistin, the serum levels of resistin were detected using ELISA. First, the expression of resistin increased in patients with GOLD stages 1 and 2 compared to patients with GOLD stages 3 and 4 (Figure 1A). Next, we analyzed the relationship between the serum resistin level and pulmonary function, which showed that the serum resistin level was negatively correlated with the forced expiratory volume in the first second percentage predicted (FEV_1_%predicted; *r* = −0.193, *p* = 0.037) and forced expiratory volume in the first second/forced vital capacity percentage predicted (FEV_1_/FVC, *r* = −0.206, *p* = 0.026; Figure 1B). The mMRC and CAT scores are widely used to assess the clinical symptoms of patients with COPD. As shown in Figure 1C, the serum resistin levels were positively correlated with the mMRC (*r* = 0.288, *p* = 0.002) and CAT scores (*r* = 0.215, *p* = 0.023). Reduced exercise tolerance is one of the main clinical characteristics of patients with COPD and is associated with a poor prognosis. The 6MWD (6-min walking distance) is a widely used tool to assess exercise tolerance. As shown in Figure 1D, the serum resistin levels were negatively correlated with the 6MWD (*r* = −0.527, *p* < 0.001). In addition, malnutrition has negative effects on lung function, as well as an increase in exacerbations and mortality. Body mass index (BMI), body fat ratio, and fat-free mass index (FFMI) are used to assess nutritional status in COPD patients. As shown in Figure 1E, the serum resistin levels were negatively correlated with BMI (*r* = −0.274, *p* = 0.003) and FFMI (*r* = −0.284, *p* = 0.014) but unrelated to the body fat ratio. Overall, these results suggested that resistin is an important biomarker in patients with COPD.

### Relationship of the Serum Resistin Level With Skeletal Muscle Function in Patients With COPD

**Figure 2:**
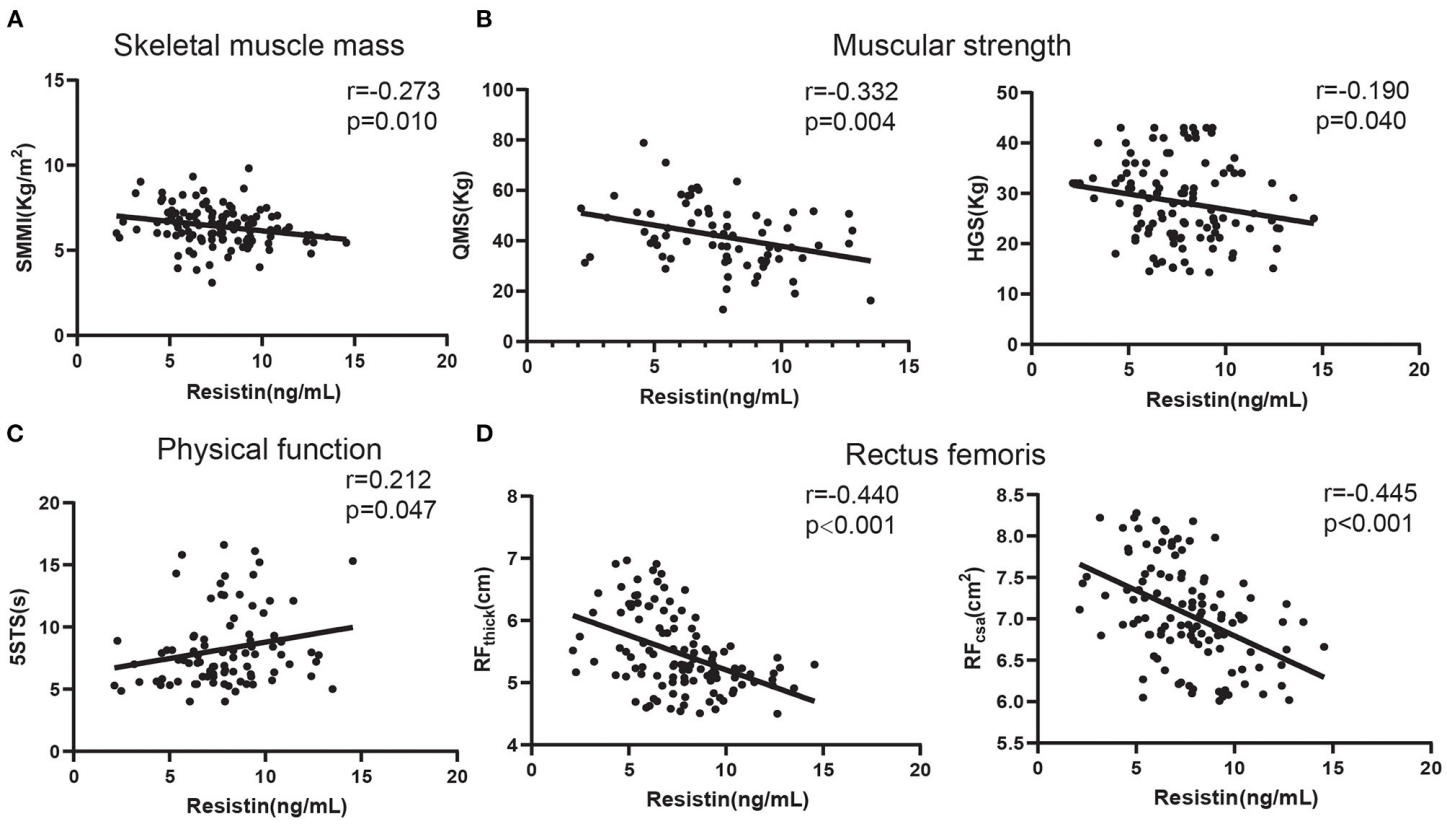
The relationships of serum resistin levels with skeletal muscle function in patients with COPD. **(A)** The relationship between serum resistin levels and SMMI; **(B)** The relationship between serum resistin levels and QMS and HGS; **(C)** The relationship between serum resistin levels and 5STS; **(D)** The relationship between serum resistin levels and RF_thick_ and RF_csa_.

Next, we analyzed the relationship between skeletal muscle function and serum resistin levels in patients with COPD. As shown in Figure 2A, the serum resistin levels were negatively correlated with the SMMI (skeletal muscle mass index; *r* = −0.273, *p* = 0.010). Next, we analyzed the relationship between resistin levels and muscular strength. As shown in Figure 2B, the serum resistin levels were negatively correlated with quadriceps muscular strength (QMS; *r* = −0.332, *p* = 0.004) and handgrip strength (HGS; *r* = −0.190, *p* = 0.040). Reduced physical performance is a common feature of patients with COPD and is caused by poor skeletal muscle function. The 5STS is used to assess physical performance; a higher 5STS indicates lower physical performance (43). As shown in Figure 2C, the serum resistin levels were positively correlated with the 5STS (*r* = 0.212, *p* = 0.047). Finally, we analyzed the relationship between resistin and the rectus femoris. An abnormal muscle quantity in the lower limbs is a manifestation of skeletal muscle dysfunction in patients with COPD (44). The thickness and cross-sectional area of the rectus femoris in patients with COPD were significantly reduced compared with those in healthy controls (45). Based on Figure 2D, the serum resistin levels were negatively correlated with RF_thick_ (*r* = −0.440, *p* < 0.001) and RF_csa_ (*r* = −0.445, *p* < 0.001). These findings suggest that resistin is an important skeletal muscle biomarker in patients with COPD.

### Clinical Factors Associated With Sarcopenia in Patients With COPD

Univariate and multivariate logistic analyses were used to explore the clinical factors associated with sarcopenia (Table 2). The results of univariate analysis showed that old age [odds ratio (OR): 1.084, 95% CI: 1.028–1.144, *p* = 0.003], high BMI (OR: 0.844, 95% CI: 0.752–0.948, *p* = 0.004), high FEV_1_/FVC (OR: 0.957, 95% CI: 0.920–0.995, *p* = 0.028), and resistin (OR: 5.147, 95% CI: 3.934–11.584, *p* < 0.001) were associated with sarcopenia in patients with COPD. Multivariate analysis showed that old age (OR: 1.115, 95% CI: 1.032–1.234, *p* = 0.008), high BMI (OR: 0.800, 95% CI: 0.683–0.937, *p* = 0.006), high FEV_1_/FVC (OR: 0.947, 95% CI: 0.912–0.987, *p* = 0.031), and serum resistin level (OR: 4.417, 95% CI: 1.645–5.029, *p* = 0.007) were independent factors.

**Table 2:** Clinical factors associated with sarcopenia in patients with COPD.

**Variable**	**Sarcopenia**			
	**Univariate analysis**		**Multivariate analysis**	
	**OR (95%CI)**	***P*-value**	**OR (95%CI)**	***P*-value**
Age	1.084 (1.028–1.144)	0.003	1.115 (1.032–1.234)	0.008
Sex	0.588 (0.255–1.357)	0.214		
BMI	0.844 (0.752–0.948)	0.004	0.800 (0.683–0.937)	0.006
FEV_1_/FVC	0.957 (0.920–0.995)	0.028	0.947 (0.912–0.987)	0.031
Serum resistin level	5.147 (3.934–11.584)	<0.001	4.417 (1.645–5.029)	0.007

### Clinical Value of Serum Resistin Levels for Predicting Sarcopenia in Patients With COPD

**Figure 3:**
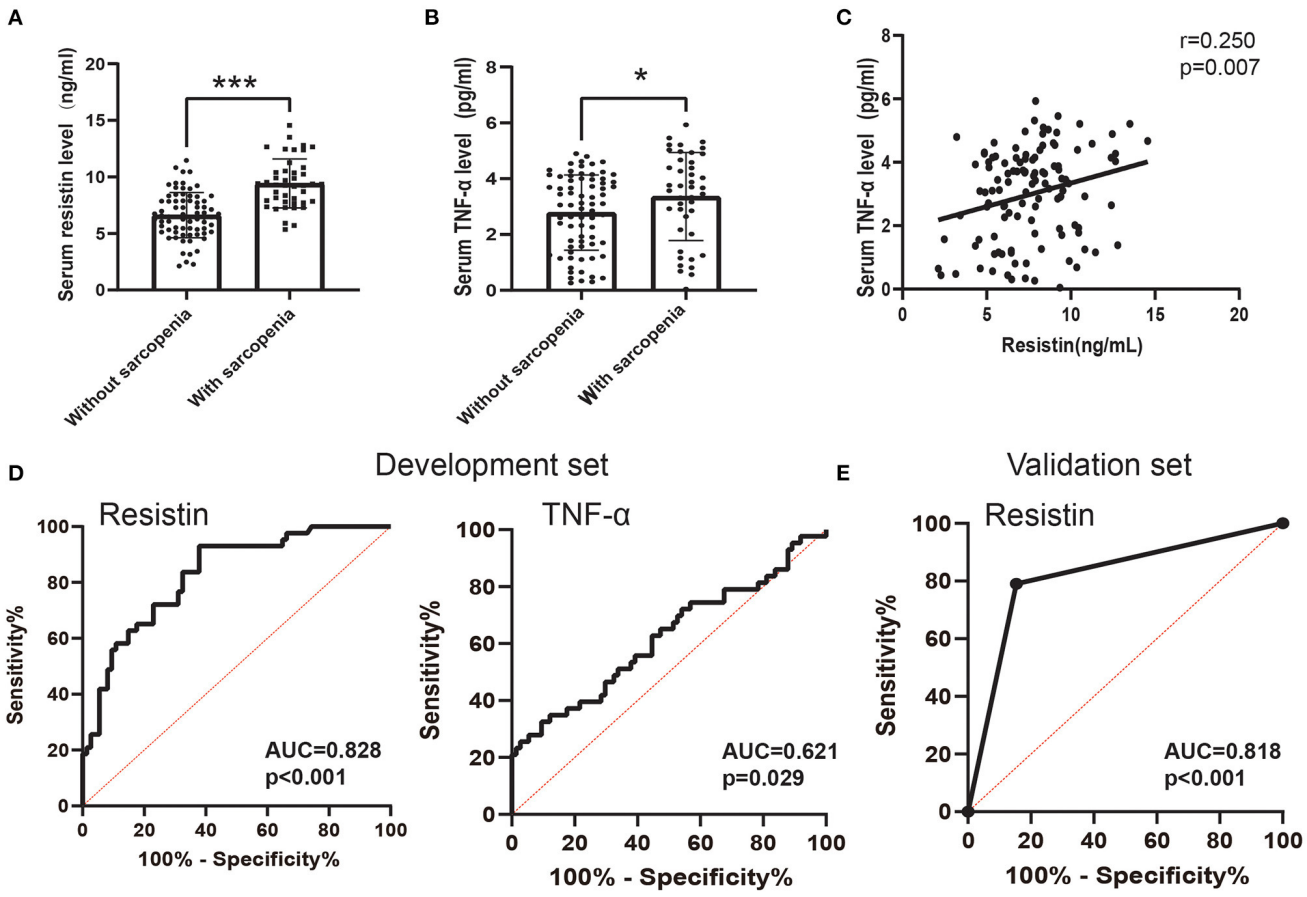
The predictive value of the serum resistin level. **(A)** Serum resistin levels differed between patients with sarcopenia and patients without sarcopenia; **(B)** Serum TNF-α levels differed between patients with sarcopenia and patients without sarcopenia; **(C)** Relationship between serum resistin levels and serum TNF-α levels; **(D)** Receiver operating characteristic curve analysis of serum resistin levels and serum TNF-α levels for the prediction of sarcopenia in the development set; **(E)** Receiver operating characteristic curve analysis of serum resistin levels for the prediction of sarcopenia in the development set. *p < 0.05, ***p < 0.001.

Previous studies indicated that a systemic inflammatory factor (TNF-α) was associated with sarcopenia in patients with COPD (20), and resistin is involved in the activation of multiple inflammatory signaling pathways (46). Therefore, we next analyzed the relationship between resistin and TNF-α expression in patients with COPD and the predictive value of the resistin level for sarcopenia. The serum levels of resistin and TNF-α were significantly higher in patients with sarcopenia than in patients without sarcopenia (Figures 3A,B). Furthermore, the serum levels of resistin were shown to be positively correlated with the serum levels of TNF-α (*r* = 0.250, *p* = 0.007) (Figure 3C). The sensitivity and specificity of the serum level of resistin for predicting sarcopenia were 93.02 and 62.16%, respectively [the cutoff point was 7.138 ng/ml, and the AUC value was 0.828 (95% CI: 0.753–0.903)] (Figure 3D). In addition, the sensitivity and specificity of the serum level of TNF-α for predicting sarcopenia were 74.42 and 47.3%, respectively [the cutoff point was 2.637 ng/ml, and the AUC value was 0.621 (95% CI: 0.511–0.731)] (Figure 3D). Because the diagnostic efficacy of resistin was significantly better than that of TNF-α, we next validated the diagnostic efficacy of resistin in the validation set. As shown in Figure 3E, the AUC value for predicting sarcopenia based on resistin was 0.818 (95% CI: 0.711–0.926, *p* < 0.001). Collectively, these results suggest that resistin may be associated with systemic inflammation and that serum resistin expression may serve as a biomarker for predicting sarcopenia in patients with COPD.

## Discussion

This is the first study to identify resistin for predicting sarcopenia in patients with COPD. In addition, we determined the cutoff value of serum resistin level that distinguished sarcopenia and validated the cutoff value in an independent validation set. Previous studies (47, 48) identified several biomarkers to predict sarcopenia in patients with COPD. Compared to these biomarkers, the strengths of this study were the large sample size, the high predictive power of serum resistin levels, and the validation of an external independent data set.

Resistin is a hormone expressed and secreted by immune cells, especially macrophages, and is associated with inflammatory responses (49). Extensive studies have shown that resistin is a key inflammatory cytokine involved in many chronic diseases, such as atherosclerosis (25), tumors (50), and diabetic vascular disease (51). We found that the serum levels of resistin were increased with increasing GOLD stage in patients with COPD and significantly correlated with pulmonary function, clinical symptoms, nutritional status, and exercise tolerance. The data reported in this study are consistent with previous reports (30, 52). The serum resistin level did not correlate with the body fat ratio in this study. In the rodent model, resistin was initially suggested to be related to the increase in fat mass, which was due to resistin being derived almost exclusively from adipose tissue in rodents (53, 54). However, resistin is expressed primarily in inflammatory cells (monocytes, macrophages, etc.) in humans and play an important role in inflammation (55, 56). BMI (weight adjusted for stature, kg/m^2^) is used to describe underweight, normal, overweight and three classes of obesity, as defined by the World Health Organization (WHO) (57). Although BMI is a good marker of overall body fat content (58, 59), it cannot discern the composition of body mass, which is defined as the proportions and distribution of bone, muscle and fat tissues in the human body. In this study, we found that serum resistin levels were associated with BMI and did not correlate with body fat ratio. This difference may be due to the serum resistin level being associated with FFMI, which represents the lean component (muscle, bone, etc.) of the body devoid of fat. Importantly, this study showed, for the first time, that serum resistin levels were negatively correlated with skeletal muscle mass and function in patients with COPD. In addition, the serum resistin levels correlated with systemic inflammation, as indicated by the serum TNF-α level, in patients. TNF-α is an important pro-inflammatory cytokine (60). The elevated expression of TNF-α in muscle or circulatory system is associated with loss of muscle strength and muscle mass (61). TNF-α leads to the degradation of skeletal muscle protein, and leads to an impaired muscle regenerative capacity. Mechanistically, the activation of NF-κB by TNF-α causes the loss of MyoD mRNA, and MyoD and myogenin are degraded *via* ubiquitin–proteasome pathway and autophagy system. In additional, TNF-α could bound to TNFR, leading to downstream activation of the caspase cascade (caspase-8 and−10) which stimulates nuclear apoptosis of skeletal muscle. Overall, TNF-α was implicated in the pathogenesis of sarcopenia. In conclusion, this study suggested that resistin is a potential biological marker in patients with COPD.

Resistin, as an adipokine, affects skeletal muscle mass or function in a variety of ways, such as *via* endocrine, inflammation, and energy metabolism (26, 62). A previous study (63) found that resistin levels are inversely correlated with muscle mass and quadricep strength. Resistin inhibits the differentiation of skeletal muscle satellite cells into skeletal muscle cells and promotes differentiation into adipocytes, leading to a decrease in skeletal muscle mass (64). Resistin effects are mediated by the Toll-like receptor 4 (TLR4) receptor, resulting in the activation of the p38 mitogen-activated protein kinase (MAPK), and nuclear factor-κB (NF-κB) pathways, involving in the occurrence and development process of muscle atrophy and muscle dysfunction. In additional, resistin induce ectopic deposition of lipids in mouse skeletal muscle cell C2C12 through miR-696 (65). Ectopic fat deposition of skeletal muscle has been recognized as an important component of sarcopenia (66, 67). These results suggest that resistin level is closely associated with the development of sarcopenia. Another study involving 2,000 subjects based on abdominal CT images showed that abdominal muscle density was negatively correlated with resistin levels and that targeted exercise of abdominal muscles helped prevent the development of sarcopenia (68). Whether resistin levels predict sarcopenia has not been established. Our results from the development and validation sets indicate that higher serum resistin levels (>7.138 ng/ml) have good predictive ability for sarcopenia. This study was the first to clarify the predictive value of the serum resistin level for sarcopenia in patients with COPD. Importantly, we determined the cutoff point of the serum resistin level, which will contribute to clinical application.

In this study, we identified TNF-α and resistin as predictors of sarcopenia in patients with COPD. And, the predictive efficacy of resistin was significantly better than that of TNF-α. TNF-α and resistin are pro-inflammatory cytokines (69, 70). Chronic systemic inflammation has been postulated as a mechanism in the loss of skeletal muscle in COPD. TNF-α has been recognized as systemic inflammatory molecules acting synchronously on lung parenchyma and the skeletal muscle in lung disease, is a key cytokine in the regulation of muscle mass signaling *via* a number of pathways (71, 72). Unlike other inflammatory factors, resistin is an adipokine. Resistin can be involved in lipid ectopic deposition by regulating fatty acid metabolism in skeletal muscle cells (73). Fatty infiltrations in skeletal muscles, resulting in decreased overall strength and functionality, leading occurrence of sarcopenia (74). This may represent the reason for the better predictive efficacy of Resistin in sarcopenia.

It is time-consuming and difficult to assess sarcopenia in all COPD patients in clinical practice, especially in community hospitals. More patients could be identified as having sarcopenia based on our prediction model, which would allow clinicians to detect and intervene with more patients at risk of sarcopenia. Therefore, serum biomarker may help clinicians diagnose sarcopenia at early admission, prehospital admission, or in the community. Sarcopenia is an age-related disease, and with the aging of the human body, there is inevitably a gradual decline in muscle mass, quality, and strength (75). In this study, we demonstrated that the association of serum resistin levels with sarcopenia in patients with COPD was independent of age. In the future, we will try to analyze the temporality of the variables and the order of occurrence of each variable through a multicenter, prospective longitudinal study.

COPD is often combined with other chronic inflammatory diseases [such as cardiovascular disease (76) and asthma (52)], which may influence serum resistin levels. In addition, resistin levels were significantly increased in patients with acute exacerbation COPD (30). Therefore, the subjects of this study were patients with stable COPD and patients with clear comorbidities (presence of severe cardiovascular disease, active lung disease, etc.) were excluded. In the future, we will try to collect more clinical samples through multicenter, prospective longitudinal studies to further analyze the clinical value of serum resistin levels in patients with different comorbidities and different disease states of COPD.

This study also had several limitations. The main limitation of this study is that our study employed a cross-sectional design, and assessments were implemented only at the time of study entry. We need to consider the fluctuations in muscle mass and resistin levels over time. Additional prospective studies are needed to support the use of resistin levels as a predictor of risk for developing sarcopenia. The BIA that we used to measure muscle mass is widely used for the diagnosis of sarcopenia but is not a good surrogate of skeletal muscle mass. In addition, the study was limited to stable COPD patients, and whether the findings can be applied to patients with acute exacerbations of COPD and those undergoing pulmonary rehabilitation is not known.

In conclusion, serum resistin levels were shown to be associated with systemic inflammation and can be used accurately and easily to predict sarcopenia in patients with COPD.

## Data Availability Statement

The original contributions presented in the study are included in the article/Supplementary Material, further inquiries can be directed to the corresponding author.

## Ethics Statement

The studies involving human participants were reviewed and approved by Research Ethics Committee of China Medical University. The patients/participants provided their written informed consent to participate in this study.

## Author Contributions

Conceptualization, writing—original draft, and writing—review and editing: GH and MD. Data curation: MD, JG, YL, YY, and XZ. Methodology: QZ. Project administration: XZ. All authors contributed to the article and approved the submitted version.

## Funding

This research was supported by National High Level Hospital Clinical Research Funding (2022-NHLHCRF-LX-01), the Non-profit Central Research Institute Fund of Chinese Academy of Medical Sciences (No. 2020-PT320-001), National Natural Science Foundation of China (No. 81900040), Liaoning Education Ministry Supporting Foundation (No. QN2019014), and Liaoning Science and Technology Ministry Supporting Foundation (No. 2019-ZD-0766).

## Conflict of Interest

The authors declare that the research was conducted in the absence of any commercial or financial relationships that could be construed as a potential conflict of interest.

## Publisher's Note

All claims expressed in this article are solely those of the authors and do not necessarily represent those of their affiliated organizations, or those of the publisher, the editors and the reviewers. Any product that may be evaluated in this article, or claim that may be made by its manufacturer, is not guaranteed or endorsed by the publisher.

## Supplementary Material

Supplementary Figure 1 The histograms **(A)**, Q-Q plots **(B)** of serum resistin data.

## References

[1] Vanfleteren LEGW Spruit MA Wouters EFM Franssen FME. Management of chronic obstructive pulmonary disease beyond the lungs. Lancet Respir Med. (2016) 4:911–24. 10.1016/S2213-2600(16)00097-7 27264777

[2] Barnes PJ. Inflammatory mechanisms in patients with chronic obstructive pulmonary disease. J Allergy Clin Immunol. (2016) 138:16–27. 10.1016/j.jaci.2016.05.011 27373322

[3] Sepúlveda-Loyola W Osadnik C Phu S Morita AA Duque G Probst VS. Diagnosis, prevalence, and clinical impact of sarcopenia in Copd: a systematic review and meta-analysis. J Cachexia Sarcopenia Muscle. (2020) 11:1164–76. 10.1002/jcsm.12600 32862514 PMC7567149

[4] Jones SE Maddocks M Kon SSC Canavan JL Nolan CM Clark AL. Sarcopenia in Copd: prevalence, clinical correlates and response to pulmonary rehabilitation. Thorax. (2015) 70:213–8. 10.1136/thoraxjnl-2014-206440 25561517

[5] Cebron Lipovec N Schols AMWJ van den Borst B Beijers RJHCG Kosten T Omersa D. Sarcopenia in advanced Copd affects cardiometabolic risk reduction by short-term high-intensity pulmonary rehabilitation. J Am Med Dir Assoc. (2016) 17:814–20. 10.1016/j.jamda.2016.05.002 27321867

[6] Sergi G Coin A Marin S Vianello A Manzan A Peruzza S. Body composition and resting energy expenditure in elderly male patients with chronic obstructive pulmonary disease. Respir Med. (2006) 100:1918–24. 10.1016/j.rmed.2006.03.008 16635565

[7] Pothirat C Chaiwong W Phetsuk N Liwsrisakun C Bumroongkit C Deesomchok A. The relationship between body composition and clinical parameters in chronic obstructive pulmonary disease. J Med Assoc Thai. (2016) 99:386–93. 10.1016/j.clnu.2021.09.035 27396222

[8] de Blasio F Di Gregorio A de Blasio F Bianco A Bellofiore B Scalfi L. Malnutrition and sarcopenia assessment in patients with chronic obstructive pulmonary disease according to international diagnostic criteria, and evaluation of raw bia variables. Respir Med. (2018) 134:1–5. 10.1016/j.rmed.2017.11.006 29413494

[9] Lee D-W Jin H-J Shin K-C Chung J-H Lee H-W Lee K-H. Presence of sarcopenia in asthma-Copd overlap syndrome may be a risk factor for decreased bone-mineral density, unlike asthma: Korean national health and nutrition examination survey (Knhanes) Iv and V (2008-2011). Int J Chron Obstruct Pulmon Dis. (2017) 12:2355–62. 10.2147/COPD.S138497 28848336 PMC5557102

[10] Cruz-Jentoft AJ Bahat G Bauer J Boirie Y Bruyère O Cederholm T. Sarcopenia: revised European consensus on definition and diagnosis. Age Ageing. (2019) 48:16–31. 10.1093/ageing/afy169 30312372 PMC6322506

[11] Chen L-K Woo J Assantachai P Auyeung T-W Chou M-Y Iijima K. Asian working group for sarcopenia: 2019 consensus update on sarcopenia diagnosis and treatment. J Am Med Dir Assoc. (2020) 21:12. 10.1016/j.jamda.2019.12.012 32033882

[12] Chung KF Adcock IM. Multifaceted mechanisms in Copd: inflammation, immunity, and tissue repair and destruction. Eur Respir J. (2008) 31:1334–56. 10.1183/09031936.00018908 18515558

[13] Decramer M Janssens W. Chronic obstructive pulmonary disease and comorbidities. Lancet Respir Med. (2013) 1:73–83. 10.1016/S2213-2600(12)70060-7 24321806

[14] Webster JM Kempen LJAP Hardy RS Langen RCJ. Inflammation and skeletal muscle wasting during cachexia. Front Physiol. (2020) 11:597675. 10.3389/fphys.2020.597675 33329046 PMC7710765

[15] MacNee W. Systemic inflammatory biomarkers and co-morbidities of chronic obstructive pulmonary disease. Ann Med. (2013) 45:291–300. 10.3109/07853890.2012.732703 23110517

[16] Schaap LA Pluijm SM Deeg DJ Harris TB Kritchevsky SB Newman AB. Higher inflammatory marker levels in older persons: associations with 5-year change in muscle mass and muscle strength. J Gerontol A Biol Sci Med Sci. (2009) 64:1183–9. 10.1093/gerona/glp097 19622801 PMC2759573

[17] Coletti D Moresi V Adamo S Molinaro M. Sassoon D. Tumor necrosis factor-A gene transfer induces cachexia and inhibits muscle regeneration. Genesis. (2005) 43:120–8. 10.1002/gene.20160 16158413

[18] Bian A-L Hu H-Y Rong Y-D Wang J Wang J-X Zhou X-ZJ. A study on relationship between elderly sarcopenia and inflammatory factors Il-6 and Tnf-n. Eur J Med Res. (2017) 22:1–8. 10.1186/s40001-017-0266-9 28701179 PMC5508730

[19] Wang Y Welc SS Wehling-Henricks M Tidball JG. Myeloid cell-derived tumor necrosis factor-alpha promotes sarcopenia and regulates muscle cell fusion with aging muscle fibers. Aging Cell. (2018) 17:e12828. 10.1111/acel.12828 30256507 PMC6260911

[20] Byun MK Cho EN Chang J Ahn CM Kim HJ. Sarcopenia correlates with systemic inflammation in Copd. Int J Chron Obstruct Pulmon Dis. (2017) 12:669–75. 10.2147/COPD.S130790 28255238 PMC5325093

[21] Ferrari R Caram LMO Faganello MM Sanchez FF Tanni SE Godoy I. Relation between systemic inflammatory markers, peripheral muscle mass, and strength in limb muscles in stable Copd patients. Int J Chron Obstruct Pulmon Dis. (2015) 10:1553–8. 10.2147/COPD.S85954 26345641 PMC4531022

[22] Kim KH Lee K Moon YS Sul HS. A cysteine-rich adipose tissue-specific secretory factor inhibits adipocyte differentiation. J Biol Chem. (2001) 276:11252–6. 10.1074/jbc.C100028200 11278254

[23] Li Y Yang Q Cai D Guo H Fang J Cui H. Resistin, a novel host defense peptide of innate immunity. Front Immunol. (2021) 12:699807. 10.3389/fimmu.2021.699807 34220862 PMC8253364

[24] Pine GM Batugedara HM Nair MG. Here, there and everywhere: resistin-like molecules in infection, inflammation, and metabolic disorders. Cytokine. (2018) 110:442–51. 10.1016/j.cyto.2018.05.014 29866514 PMC6103837

[25] Zhou L Li J-Y He P-P Yu X-H Tang C-K. Resistin: potential biomarker and therapeutic target in atherosclerosis. Clin Chim Acta. (2021) 512:84–91. 10.1016/j.cca.2020.11.010 33248946

[26] O'Leary MF Wallace GR Davis ET Murphy DP Nicholson T Bennett AJ. Obese subcutaneous adipose tissue impairs human myogenesis, particularly in old skeletal muscle, *via* resistin-mediated activation of Nfκb. Sci Rep. (2018) 8:15360. 10.1038/s41598-018-33840-x 30337633 PMC6193975

[27] Priego T Martín AI González-Hedström D Granado M López-Calderón A. Role of hormones in sarcopenia. Vitam Horm. (2021) 115:535–70. 10.1016/bs.vh.2020.12.021 33706961

[28] Jørgensen SB Honeyman J Oakhill JS Fazakerley D Stöckli J Kemp BE. Oligomeric resistin impairs insulin and aicar-stimulated glucose uptake in mouse skeletal muscle by inhibiting Glut4 translocation. Am J Physiol Endocrinol Metab. (2009) 297:E57–66. 10.1152/ajpendo.90744.2008 19435854

[29] Barclay RD Burd NA Tyler C Tillin NA Mackenzie RW. The role of the Igf-1 signaling cascade in muscle protein synthesis and anabolic resistance in aging skeletal muscle. Front Nutr. (2019) 6:146. 10.3389/fnut.2019.00146 31552262 PMC6746962

[30] Kumor-Kisielewska A Kierszniewska-Stepień D Pietras T Kroczyńska-Bednarek J Kurmanowska Z Antczak A. Assessment of leptin and resistin levels in patients with chronic obstructive pulmonary disease. Pol Arch Med Wewn. (2013) 123:215–20. 10.20452/pamw.1724 23611920

[31] Vondracek SF Voelkel NF McDermott MT Valdez C. The relationship between adipokines, body composition, and bone density in men with chronic obstructive pulmonary disease. Int J Chron Obstruct Pulmon Dis. (2009) 4:267–77. 10.2147/COPD.S2745 19657401 PMC2719257

[32] Halpin DMG Criner GJ Papi A Singh D Anzueto A Martinez FJ. Global initiative for the diagnosis, management, and prevention of chronic obstructive lung disease. the 2020 Gold science committee report on covid-19 and chronic obstructive pulmonary disease. Am J Respir Crit Care Med. (2021) 203:24–36. 10.1164/rccm.202009-3533SO 33146552 PMC7781116

[33] Peduzzi P Concato J Kemper E Holford TR Feinstein AR. A simulation study of the number of events per variable in logistic regression analysis. J Clin Epidemiol. (1996) 49:1373–9. 10.1016/S0895-4356(96)00236-3 8970487

[34] Lage VKdS de Paula FA Dos Santos JM Costa HS da Silva GP Lima LP. Are oxidative stress biomarkers and respiratory muscles strength associated with copd-related sarcopenia in older adults? Exp Gerontol. (2022) 157:111630. 10.1016/j.exger.2021.111630 34813902

[35] Buderer NM. Statistical methodology: I. Incorporating the prevalence of disease into the sample size calculation for sensitivity and specificity. Acad Emerg Med. (1996) 3:895–900. 10.1111/j.1553-2712.1996.tb03538.x 8870764

[36] Bestall JC Paul EA Garrod R Garnham R Jones PW Wedzicha JA. Usefulness of the medical research council (Mrc) dyspnoea scale as a measure of disability in patients with chronic obstructive pulmonary disease. Thorax. (1999) 54:581–6. 10.1136/thx.54.7.581 10377201 PMC1745516

[37] Jones PW Harding G Berry P Wiklund I Chen WH Kline Leidy N. Development and first validation of the Copd assessment test. Eur Respir J. (2009) 34:648–54. 10.1183/09031936.00102509 19720809

[38] ATS Committee on Proficiency Standards for Clinical Pulmonary Function Laboratories. Ats statement: guidelines for the six-minute walk test. Am J Respir Crit Care Med. (2002) 166:111–7. 10.1164/ajrccm.166.1.at1102 12091180

[39] Maynard-Paquette A-C Poirier C Chartrand-Lefebvre C Dubé B-P. Ultrasound evaluation of the quadriceps muscle contractile index in patients with stable chronic obstructive pulmonary disease: relationships with clinical symptoms, disease severity and diaphragm contractility. Int J Chron Obstruct Pulmon Dis. (2020) 15:79–88. 10.2147/COPD.S222945 32021146 PMC6957010

[40] Deng M Liang C Yin Y Shu J Zhou X Wang Q. Ultrasound assessment of the rectus femoris in patients with chronic obstructive pulmonary disease predicts poor exercise tolerance: an exploratory study. BMC Pulm Med. (2021) 21:304. 10.1186/s12890-021-01663-8 34563152 PMC8466975

[41] Deng M Zhou X Li Y Yin Y Liang C Zhang Q. Ultrasonic elastography of the rectus femoris, a potential tool to predict sarcopenia in patients with chronic obstructive pulmonary disease. Front Physiol. (2021) 12:783421. 10.3389/fphys.2021.783421 35069243 PMC8766419

[42] Li X-L Wu C Xie J-G Zhang B Kui X Jia D. Development and validation of a nomogram for predicting the disease progression of nonsevere coronavirus disease 2019. J Transl Int Med. (2021) 9:131–42. 10.2478/jtim-2021-0030 34497752 PMC8386326

[43] Lage VKS Silva GP Lacerda ACR Paula FA Lima LP Santos JNV. Functional tests associated with sarcopenia in moderate chronic obstructive pulmonary disease. Expert Rev Respir Med. (2021) 15:569–76. 10.1080/17476348.2021.1850276 33197358

[44] Man WDC Soliman MGG Nikoletou D Harris ML Rafferty GF Mustfa N. Non-volitional assessment of skeletal muscle strength in patients with chronic obstructive pulmonary disease. Thorax. (2003) 58:665–9. 10.1136/thorax.58.8.665 12885979 PMC1746754

[45] Shrikrishna D Patel M Tanner RJ Seymour JM Connolly BA Puthucheary ZA. Quadriceps wasting and physical inactivity in patients with Copd. Eur Respir J. (2012) 40:1115–22. 10.1183/09031936.00170111 22362854

[46] Jang JC Li J Gambini L Batugedara HM Sati S Lazar MA. Human resistin protects against endotoxic shock by blocking Lps-Tlr4 interaction. Proc Natl Acad Sci U S A. (2017) 114:E10399–E408. 10.1073/pnas.1716015114 29133417 PMC5715788

[47] Qaisar R Karim A Muhammad T Shah I Khan J. Prediction of sarcopenia using a battery of circulating biomarkers. Sci Rep. (2021) 11:8632. 10.1038/s41598-021-87974-6 33883602 PMC8060253

[48] Hirai K Tanaka A Homma T Goto Y Akimoto K Uno T. Serum creatinine/cystatin C ratio as a surrogate marker for sarcopenia in patients with chronic obstructive pulmonary disease. Clin Nutr. (2021) 40:1274–80. 10.1016/j.clnu.2020.08.010 32863062

[49] Taouis M Benomar Y. Is resistin the master link between inflammation and inflammation-related chronic diseases? Mol Cell Endocrinol. (2021) 533:111341. 10.1016/j.mce.2021.111341 34082045

[50] Sudan SK Deshmukh SK Poosarla T Holliday NP Dyess DL Singh AP. Resistin: an inflammatory cytokine with multi-faceted roles in cancer. Biochim Biophys Acta Rev Cancer. (2020) 1874:188419. 10.1016/j.bbcan.2020.188419 32822824 PMC8117252

[51] Biscetti F Nardella E Cecchini AL Flex A Landolfi R. Biomarkers of vascular disease in diabetes: the adipose-immune system cross talk. Intern Emerg Med. (2020) 15:381–93. 10.1007/s11739-019-02270-6 31919781

[52] Al Mutairi SS Mojiminiyi OA Shihab-Eldeen A Al Rammah T Abdella N. Putative roles of circulating resistin in patients with asthma, Copd and cigarette smokers. Dis Markers. (2011) 31:1–7. 10.1155/2011/297591 21846943 PMC3826866

[53] Steppan CM Bailey ST Bhat S Brown EJ Banerjee RR Wright CM. The hormone resistin links obesity to diabetes. Nature. (2001) 409:307–12. 10.1038/35053000 11201732

[54] Steppan CM Brown EJ Wright CM Bhat S Banerjee RR Dai CY. A family of tissue-specific resistin-like molecules. Proc Natl Acad Sci U S A. (2001) 98:502–6. 10.1073/pnas.98.2.502 11209052 PMC14616

[55] Patel L Buckels AC Kinghorn IJ Murdock PR Holbrook JD Plumpton C. Resistin is expressed in human macrophages and directly regulated by Ppar gamma activators. Biochem Biophys Res Commun. (2003) 300:472–6. 10.1016/S0006-291X(02)02841-3 12504108

[56] Yang R-Z Huang Q Xu A McLenithan JC Eisen JA Shuldiner AR. Comparative studies of resistin expression and phylogenomics in human and mouse. Biochem Biophys Res Commun. (2003) 310:927–35. 10.1016/j.bbrc.2003.09.093 14550293

[57] Baracos VE Arribas L. Sarcopenic obesity: hidden muscle wasting and its impact for survival and complications of cancer therapy. Ann Oncol. (2018) 29(Suppl. 2):ii1–ii9. 10.1093/annonc/mdx810 32169202

[58] Sanchez A Kissel S Coletta A Scott J Furberg H. Impact of body size and body composition on bladder cancer outcomes: risk stratification and opportunity for novel interventions. Urol Oncol. (2020) 38:713–8. 10.1016/j.urolonc.2020.03.017 32312642 PMC8245008

[59] Gonzalez MC Correia M Heymsfield SB. A requiem for Bmi in the clinical setting. Curr Opin Clin Nutr Metab Care. (2017) 20:314–21. 10.1097/MCO.0000000000000395 28768291

[60] Chao J Cui S Liu C Liu S Liu S Han Y. Detection of early cytokine storm in patients with septic shock after abdominal surgery. J Transl Int Med. (2020) 8:91–8. 10.2478/jtim-2020-0014 32983931 PMC7500114

[61] Zhang X Li H He M Wang J Wu Y Li Y. Immune system and sarcopenia: presented relationship and future perspective. Exp Gerontol. (2022) 164:111823. 10.1016/j.exger.2022.111823 35504482

[62] Fan H-Q Gu N Liu F Fei L Pan X-Q Guo M. Prolonged exposure to resistin inhibits glucose uptake in rat skeletal muscles. Acta Pharmacol Sin. (2007) 28:410–6. 10.1111/j.1745-7254.2007.00523.x 17303005

[63] Bucci L Yani SL Fabbri C Bijlsma AY Maier AB Meskers CG. Circulating levels of adipokines and Igf-1 are associated with skeletal muscle strength of young and old healthy subjects. Biogerontology. (2013) 14:261–72. 10.1007/s10522-013-9428-5 23666343

[64] Sheng CH Du ZW Song Y Wu XD Zhang YC Wu M. Human resistin inhibits myogenic differentiation and induces insulin resistance in myocytes. Biomed Res Int. (2013) 2013:804632. 10.1155/2013/804632 23509781 PMC3590612

[65] Wen F Zhang H Bao C Yang M Wang N Zhang J. Resistin increases ectopic deposition of lipids through Mir-696 in C2c12 cells. Biochem Genet. (2015) 53:63–71. 10.1007/s10528-015-9672-2 25962325

[66] Xue M Zhang F Ji X Yu H Jiang X Qiu Y. Oleate ameliorates palmitate-induced impairment of differentiative capacity in C2c12 myoblast cells. Stem Cells Dev. (2021) 30:289–300. 10.1089/scd.2020.0168 33430700

[67] Yuan F Zhang Q Dong H Xiang X Zhang W Zhang Y. Effects of Des-Acyl ghrelin on insulin sensitivity and macrophage polarization in adipose tissue. J Transl Int Med. (2021) 9:84–97. 10.2478/jtim-2021-0025 34497748 PMC8386331

[68] Van Hollebeke RB Cushman M Schlueter EF Allison MA. Abdominal muscle density is inversely related to adiposity inflammatory mediators. Med Sci Sports Exerc. (2018) 50:1495–501. 10.1249/MSS.0000000000001570 29401141 PMC6005727

[69] Semiz A Ozgun Acar O Cetin H Semiz G Sen A. Suppression of inflammatory cytokines expression with bitter melon () in Tnbs-instigated ulcerative colitis. J Transl Int Med. (2020) 8:177–87. 10.2478/jtim-2020-0027 33062594 PMC7534491

[70] Shin JH Park S Cho H Kim JH Choi H. Adipokine human resistin promotes obesity-associated inflammatory intervertebral disc degeneration *via* pro-inflammatory cytokine cascade activation. Sci Rep. (2022) 12:8936. 10.1038/s41598-022-12793-2 35624126 PMC9142523

[71] Passey SL Hansen MJ Bozinovski S McDonald CF Holland AE Vlahos R. Emerging therapies for the treatment of skeletal muscle wasting in chronic obstructive pulmonary disease. Pharmacol Ther. (2016) 166:56–70. 10.1016/j.pharmthera.2016.06.013 27373503

[72] Zeng Q Wang X-H Yang L-P Lang R Liang Y Yu R-H. Shengxuening oral iron supplementation for the treatment of renal anemia: a systematic review. J Transl Int Med. (2020) 8:245–54. 10.2478/jtim-2020-0037 33511051 PMC7805283

[73] Alizadeh Pahlavani H. Exercise therapy for people with sarcopenic obesity: myokines and adipokines as effective actors. Front Endocrinol. (2022) 13:811751. 10.3389/fendo.2022.811751 35250869 PMC8892203

[74] Ke Y Xu C Lin J Li Y. Role of hepatokines in non-alcoholic fatty liver disease. J Transl Int Med. (2019) 7:143–8. 10.2478/jtim-2019-0029 32010600 PMC6985917

[75] Jin H Xie W He M Li H Xiao W Li Y. Pyroptosis and sarcopenia: frontier perspective of disease mechanism. Cells. (2022) 11:1078. 10.3390/cells11071078 35406642 PMC8998102

[76] Karnati S Seimetz M Kleefeldt F Sonawane A Madhusudhan T Bachhuka A. Chronic obstructive pulmonary disease and the cardiovascular system: vascular repair and regeneration as a therapeutic target. Front. Cardiovasc. Med. (2021) 8:649512. 10.3389/fcvm.2021.649512 33912600 PMC8072123

