# Supplementary figures and images for: Resistin as a Systemic Inflammation-Related Biomarker for Sarcopenia in Patients With Chronic Obstructive Pulmonary Disease
Source: Front Nutr. 2022 Jul 12;9:921399. doi: 10.3389/fnut.2022.921399 (PMC9315354; doi:10.3389/fnut.2022.921399)

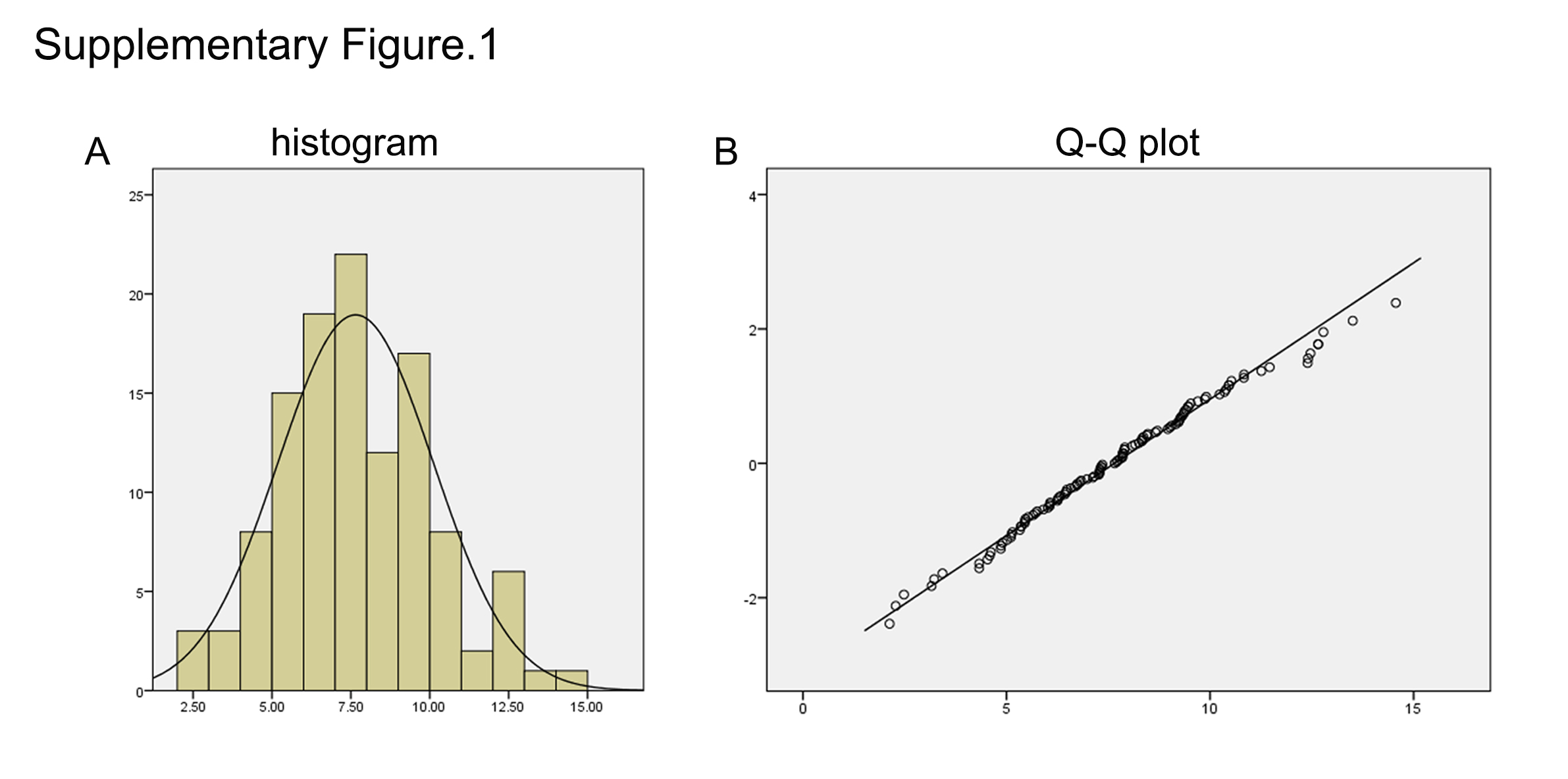

Supplement: Supplementary Figure 1 — The histograms (A), Q-Q plots (B) of serum resistin data. [file Image_1.jpeg]
